# Pleiotropic Effects of the NSAID Fenamates on Chloride Channels: Opportunity for Ion Channelopathies?

**DOI:** 10.1002/prp2.70144

**Published:** 2025-06-26

**Authors:** Paola Laghetti, Ilaria Saltarella, Simone Dell'Atti, Jean‐François Desaphy, Concetta Altamura

**Affiliations:** ^1^ Section of Pharmacology, Department of Precision and Regenerative Medicine School of Medicine, University of Bari Aldo Moro Bari Italy

**Keywords:** channelopathies, chloride channels, fenamates, NSAIDs, pharmacology

## Abstract

Chloride channels are involved in many cellular processes, including cell volume regulation, modulation of cell excitability, and electrolyte and water secretion. Mutations of these proteins are associated with heterogeneous diseases such as myotonia, cystic fibrosis, epilepsy, deafness, lysosomal storage disease, and various kinds of renal and ophthalmic dysfunctions, also known as channelopathies. Thus, drugs targeting chloride channels may have important therapeutic applications. In this context, fenamates, commonly used for their anti‐inflammatory properties, have been explored for drug repurposing in chloride channelopathies thanks to their ability to modulate multiple chloride channels. This narrative review resumes the effects of niflumic acid (NFA), flufenamic acid (FFA), mefenamic acid (MFA), meclofenamic acid (MCFA), and tolfenamic acid (TFA) on different types of chloride channel. It emerges that fenamates have a wide spectrum of activities on these channels that vary depending on multiple factors like channel isoforms, extracellular and intracellular conditions, and cell and tissue types. They may also exhibit both activating and inhibitory effects depending on their concentration. Therefore, thanks to their variegated modulatory activity on chloride channels, fenamates might be considered promising lead compounds for the development of new drug candidates that can target these altered channels involved in channelopathies.

**Trial Registration:** EudraCT number: 2021‐000708‐39; ClinicalTrials.gov identifier: NCT029930005 and NCT02429570.

## 1 Introduction

Chloride channels are pore‐forming membrane proteins that exert countless roles in human cells, including cell volume regulation, stabilization of membrane voltage and modulation of cell excitability, electrolyte, and water secretion [1]. They can be classified as members of voltage‐gated channels (ClC), calcium activated Cl^−^ channels (CaCC), cystic fibrosis transmembrane conductance regulator (CFTR), volume regulated channels, ligand‐gated anion channels (glycine and GABA_A_ receptors), and maxi‐anions channels [2]. Alteration of chloride transport is responsible for different diseases, also known as chloride channelopathies, like myotonia, cystic fibrosis, epilepsy, deafness, lysosomal storage disease, and various kinds of renal disfunctions (e.g., renal salt loss, kidney stones, and osteopetrosis), characterized by a wide range of symptoms [3]. Additionally, besides their canonical role, chloride channels have also been investigated for their involvement in cancer onset, tumor progression, as they can modulate cell proliferation, motility and fate as well as response to therapy [4, 5, 6].

Despite their crucial role in physiology and in many human diseases, chloride channels are often overlooked as drug targets compared to cation channels due to the limited availability of known modulators and to technical issues correlated to their screening [7].

**FIGURE 1:**
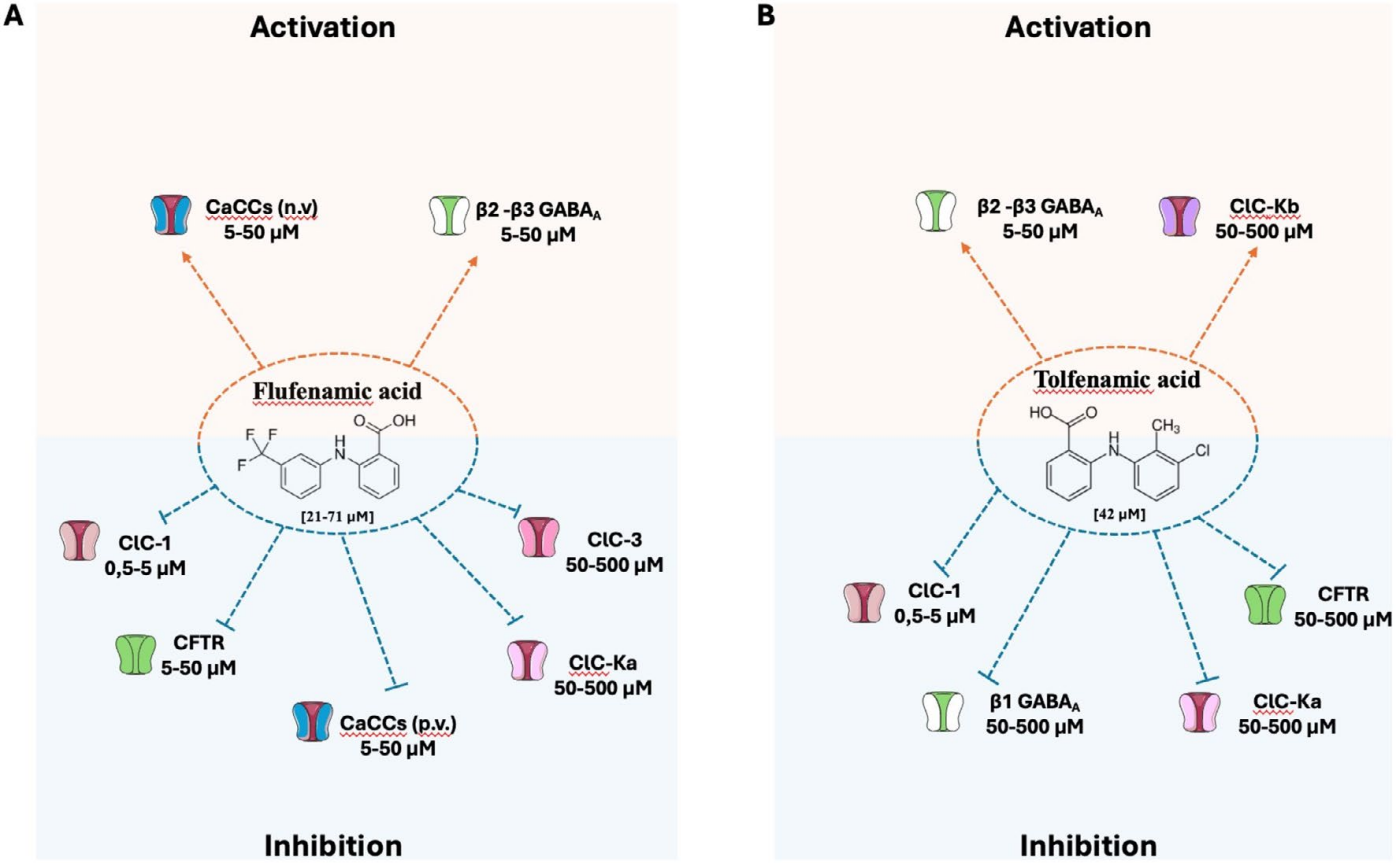
Effects of (A) flufenamic and (B) tolfenamic acid on chloride channels. These compounds belong to the fenamates, which are derivatives of anthranilic acid, characterized by a benzene ring, ortho‐substituted with a carboxylic acid and an amine. These drugs exhibit either activation or inhibitory effects on ion channels. The reported IC_50_ values or drug concentrations tested for each channel are provided. These inhibitory or activation values are not always fully comparable since they have been evaluated on different systems (heterologous or native systems, in vitro or ex vivo) by considering different effects (current amplitude, voltage dependence…). Further details, abbreviations, and references are available in the main text. n.v., negative voltage; p.v., positive voltage.

**FIGURE 2:**
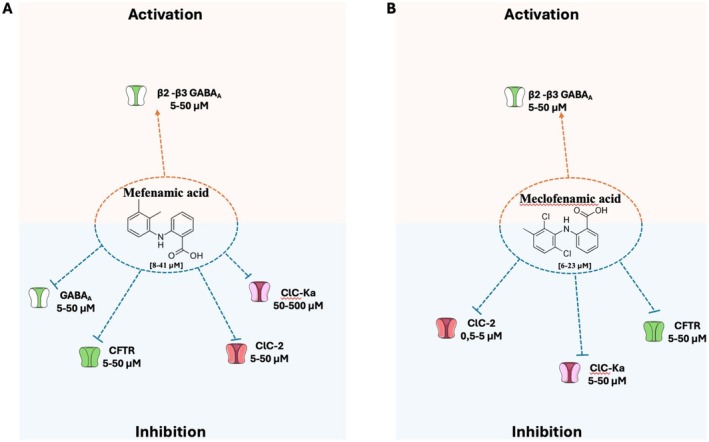
Effects of (A) mefenamic and (B) meclofenamic acid on chloride channels. These drugs exhibit either activation or inhibitory effects on ion channels. The reported IC_50_ values or drug concentrations tested for each channel are provided. These inhibitory or activation values are not always fully comparable since they have been evaluated on different systems (heterologous or native systems, in vitro or ex vivo) by considering different effects (current amplitude, voltage dependence…). Further details, abbreviations, and references are available in the main text.

**FIGURE 3:**
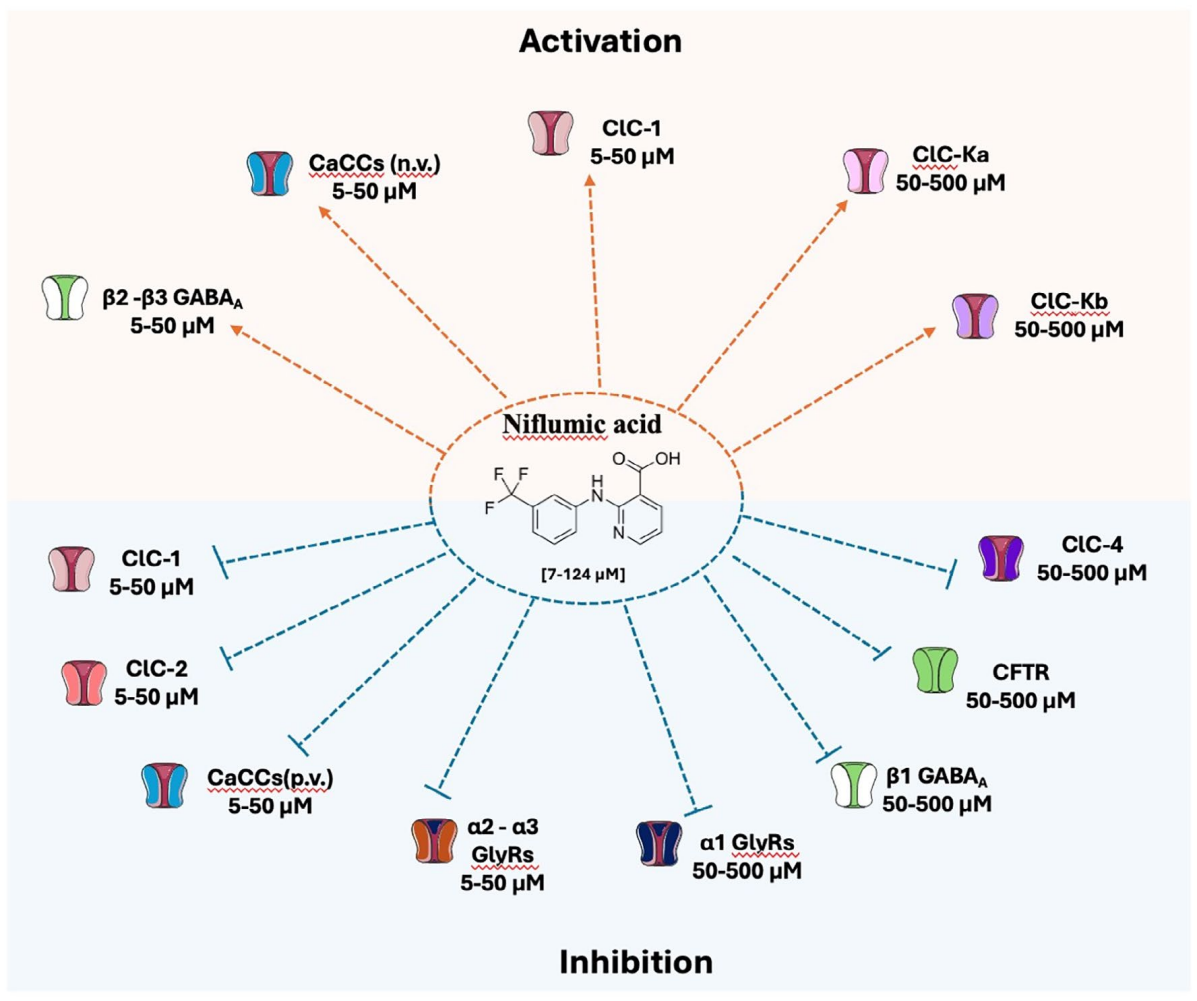
Effects of niflumic acid on chloride channels. The drug exhibits either activation or inhibitory effects on ion channels. The reported IC_50_ values or drug concentrations tested for each channel are provided. These inhibitory or activation values are not always fully comparable since they have been evaluated on different systems (heterologous or native systems, in vitro or ex vivo) by considering different effects (current amplitude, voltage dependence…). Further details, abbreviations, and references are available in the main text.

Among approved drugs, fenamates, a group of nonsteroidal anti‐inflammatory drugs (NSAIDs), exhibit modulatory effects on ion channels, including chloride channels (Figures 1, 2, 3), in addition to their canonical role in reducing inflammation by inhibiting the cyclooxygenase enzyme, which converts arachidonic acid into the inflammatory mediators thromboxanes and prostaglandins. Niflumic acid (NFA), meclofenamic acid (MCFA), flufenamic acid (FFA), tolfenamic acid (TFA) and mefenamic acid (MFA) are the fenamates currently used in clinical practice to treat moderate pain, rheumatoid arthitis, musculoskeletal pain, migraine headaches, idiopathic dysmenorrhea and neoplastic pain. As these fenamates have shown either activation or inhibitory effects on ion channels [8, 9, 10], they represent attractive candidates for targeting ion channels‐related diseases or for being the starting point for the design of new chloride channels modulators.

Hence, this review aims to review recent evidence of fenamates' ability to modulate different types of chloride channel, exploring their potential repurposing for the treatment of chloride channelopathies.

The scientific literature was searched using the following databases: Science Direct, PubMed, Google Scholar and Web of Science. Only full‐text articles were considered for writing purposes, and references included a list of the literature pertaining to the body of collected research. Further relevant articles were extracted from the reference sections of the reviewed articles. In addition, ClinicalTrials.gov and European Clinical Trials Database were used for clinical trials details.

## 2 CLC Chloride Channels and Transporters

In humans, the CLC protein family encompasses nine isoforms, of which four function as plasma membrane chloride channels (ClC‐1, ClC‐2, ClCKa, and ClCKb) and five function as intracellular chloride/proton exchangers [ClC‐3 to ClC7] [2].

ClC‐1 is a voltage‐gated channel mainly expressed in skeletal muscle cells, responsible for the sarcolemma chloride conductance involved in the stabilization of resting membrane potential and repolarization phase of action potential [11]. Loss of function mutations in the *CLCN1* gene encoding ClC‐1 cause myotonia congenita (MC) [12, 13], a rare disease characterized by sarcolemma hyper‐excitability responsible for muscle stiffness after voluntary muscle contraction, which improves with consecutive movements and worsens with inactivity [14]. The mutations can affect ClC‐1 function at different levels, altering the voltage dependence of the slow and fast gates, the intracellular trafficking, the conductance, or the ion selectivity [15]. Nowadays, no direct activators of ClC‐1 are known, and the first line for the treatment of MC is based on the use of a sodium channel blocker. Nevertheless, new potentially active molecules are emerging. The effects of niflumic acid were studied on sarcolemma chloride conductance (gCl) of rat muscle fibers and on ClC‐1 channels heterologously expressed in Sf9 cells or *Xenopus* oocytes [16, 17]. The drug exerts voltage‐dependent and reversible inhibition of ClC‐1 channels and a reduction of sarcolemma gCl with IC_50_ around 50 μM (Figure 3). NFA was also shown to increase [Ca^2+^]_i_ in rat skeletal muscle fibers with IC_50_ of ~100 μM, by stimulating Ca^2+^ release from mitochondria [17]. Accordingly, further experiments showed that part of the NFA effect on gCl in muscle fibers was mediated by PKC, which is known to inhibit ClC‐1 channels. A SAR study with NFA analogues suggested that the substitution with an electron‐attractive group in the meta position of the phenyl group is critical for ClC‐1 inhibition, while elimination of the pyridinic nitrogen, as in FFA and TFA, further increases potency. In contrast, none of the analogues were as potent as NFA in increasing [Ca^2+^]_i_ (Figure 1). Computational studies of a homology model of ClC‐1 built upon a eukaryotic CLC crystal structure revealed four putative binding cavities in ClC‐1, and docking studies suggested that NFA may bind to a small pocket, with R421 and F484 as critical residues [18]. More recently, the structure of the human ClC‐1 chloride channel was resolved using single‐particle cryo‐electron microscopy, revealing similarities to bovine ClC‐K and CLC transporters. This structural analysis provides valuable insights into the chloride transport pathway and may help validate predicted binding sites [19]. Furthermore, NFA was used to demonstrate the potential of pharmacological chaperones in myotonia congenita (MC), a rare skeletal muscle disease due to loss‐of‐function ClC‐1 channel mutations [20]. Incubation of cells expressing traffic‐defective ClC‐1 channel mutants with 50 μM NFA for 24 h restored channel membrane expression and chloride current similar to wild‐type, suggesting the therapeutic potential of fenamates for the treatment of MC patients (Figure 3).

ClC‐2 is a widespread voltage‐gated chloride channel activated by membrane hyperpolarization and cell swelling. It is involved in the regulation of excitability, cell volume, and extracellular ion homeostasis in the eye, testes, and in the digestive, nervous, respiratory, and circulatory systems [21, 22]. Loss‐of‐function mutations of ClC‐2 lead to leukoencephalopathy, visual impairment [23, 24] and male infertility [25], while gain‐of‐function mutations may cause hyperaldosteronism and cardiac arrhythmias [26, 27]. Recently, Koster et al. performed a screening of different approved drugs, including fenamates, on ClC‐2 channels expressed in Chinese hamster ovary (CHO) cells using an automated patch‐clamp platform [28]. Among these drugs, MCFA was identified as a “hit compound” showing inhibitory activity on the channel (IC_50_ ~10 μM), whereas MFA and NFA had very little effect (Figures 2 and 3). Consequently, various compounds were developed starting from the MCFA structure. Specifically, the compound named AK‐42 inhibited the ClC‐2 channel at nanomolar concentration (IC_50_ ~15 nM) and showed a great selectivity for ClC‐2 over other channels (including ClC‐1 and other anion channels), receptors, and transporters expressed in the central nervous system, supporting the potential of this compound for the study of ClC‐2 function [28]. Further molecular insight into AK‐42 binding to ClC‐2 has been recently provided [29, 30].

The ClC‐Ka and ClC‐Kb channels are expressed in renal cells and involved in transepithelial transport and in Cl^−^ exit from the basolateral membrane [2]. Specifically, ClC‐Ka is involved in Cl^−^ transport in the thin ascending limb and, in mouse, loss‐of‐function mutations of this channel are responsible for nephrogenic insipidus diabetes [31]. ClC‐Kb manages the NaCl transport in thick ascending limb and collecting ducts and genetic loss‐of‐function mutations lead to Bartter's syndrome type III in humans, characterized by hypokalemia and salt loss [32]. Fenamates were tested on human ClC‐Ka/b channels expressed in *Xenopus* oocytes [33]. NFA induced activation of ClC‐Ka channels in a bell‐shaped concentration manner, with greatest chloride current activation at ~500 μM; this suggests the presence of two functional NFA binding sites with different affinities, a high‐affinity activating site and a low‐affinity inhibitory site [33, 34] (Figure 3). Activating effect appeared to depend on the presence of the pyridinic cycle in FFA, since other fenamates (MCFA, MFA, TFA and FFA) were only inhibitory [33] (Figures 1 and 2). The pyridinic cycle confers coplanarity to FFA molecule, which is likely critical for ClC‐Ka activation [35]. Notably, recent studies demonstrated that FFA, MFA and TFA undergo conformational changes when embedded in phosphatidyloleoylphosphatidylcholine (POPC) bilayers, a model for cell membranes. Among these, FFA adopts a peculiar conformation that enhances its binding affinity to POPC compared to other fenamates. This behavior may affect the interaction with the channel binding‐site, potentially contributing to the variations in efficacy and potency observed among different fenamates [36, 37]. The most potent inhibitor was MCFA with a *K*_D_ of ~40 μM both at negative and positive voltages. The inhibitory binding site of fenamates likely overlaps that of the previously identified inhibitor, 3‐phenyl‐CPP, involving the Asn68 residue [34, 38]. In ClC‐Kb, Asn68 is substituted for by Asp68, reducing inhibitory effects of 3‐phenyl‐CPP [34] and fenamates [33] on ClC‐Kb. Consequently, at low concentrations, activation of ClC‐Kb currents by NFA was more pronounced compared to ClC‐Ka, and FFA was able to increase ClC‐Kb currents (Figures 1 and 3). Some aminoacidic residues possibly involved in the activation of ClC‐Ka by NFA were identified (L155, G345, A349) [39]. Another study suggested the importance of the I‐J loop in calcium sensitivity of ClC‐Ka, including F256 and N257 as critical residues for channel activation by NFA [40]. It is noteworthy that ClC‐Ka activation by NFA was not observed in transfected HEK cells, maybe due to basal overactivity of the channel in the mammalian cell line compared to *Xenopus* oocytes [41]. This underlines the difficulty in studying ion channel pharmacology in heterologous systems and the need for confirmatory experiments in more physiological systems. Yet, such sophisticated SAR studies may open the way for development of compounds of clinical interest, since openers of ClC‐Ka channels might prove beneficial in Bartter syndrome.

The ClC‐3, ClC‐4, and ClC‐5 proteins share ~80% amino acid sequence identity, while ClC‐6 and ClC‐7 display ~45% homology. They all function as H^+^/Cl^−^ antiports and are mostly expressed in the endosomal/lysosomal system, although expression in the endoplasmic/sarcoplasmic reticulum and at the plasma membrane has also been suggested for some of them [2]. Genetic variants have been associated with several human diseases including developmental encephalopathies (ClC‐3 and ClC‐4), Dent's disease (ClC‐5), and osteopetrosis (ClC‐7). Two reports suggested the inhibitory effect of FFA on ClC‐3 [42] and NFA on ClC‐4 [43] (Figures 1 and 3); however, confirmatory studies are required since there is a lot of confusion regarding data interpretation regarding these two exchangers. To date, no effect on fenamates has been reported for ClC‐5, 6, and 7.

## 3 Ca^2+^‐Activated Chloride Channel

Ca2+‐activated chloride channels (CaCCs) are present in different tissues and cell types with a broad range of functions such as sensory transduction, epithelial secretion, and smooth muscle contraction. Several studies demonstrated the blocking activity of fenamates NFA and FFA on different CaCCs suggesting their potential application for the modulation of several physiological and pathological processes [44, 45, 46, 47, 48, 49].

The molecular counterpart of calcium‐sensitive chloride currents has long been debated. Transmembrane protein with unknown function 16 (TMEM16A‐K, also called anoctamin 1–10), CLCA (Chloride Channel Accessory Family), and bestrophins (BEST), all have been proposed as major components of CaCCs [50]. Today, the more compelling evidence suggests that some members of the TMEM16/anoctamin family may work as calcium‐sensitive chloride channels [51, 52].

NFA was shown to block CaCCs carried by heterologously expressed TMEM16A, 16B, and 16F [53, 54, 55, 56, 57] (Figure 3). Among seven known inhibitors of TMEM16A, NFA was the most potent to inhibit CaCCs in transfected CHO cells, with an IC_50_ of ~7.5 μM followed by FFA (IC_50_ = ~14 μM) at positive voltages [58] (Figures 1 and 3). In contrast, both NFA and FFA had a dual effect at negative potential (−80 mV): they increased the inward current at lower concentrations (< 100 μM) and inhibited it at higher concentrations (> 100 μM) [58]. Another study of NFA on TMEM16A expressed in HEK cells reported similar observations [59]. Such a dual effect of NFA (activation at negative voltage and inhibition at positive voltages) had been already observed on native CaCCs in rabbit artery myocytes, suggesting the presence of two binding sites [46, 47]. The slowing of current deactivation at negative voltages by NFA and FFA might explain the observed increase in current amplitude [58, 59] (Figures 1 and 3). Interestingly, another study reported that NFA blocking activity on TMEM16A is apparently antagonized by anion occupancy of the channel pore [60]. Since the NFA binding site is not known, such an observation suggests either a competitive binding of NFA and anions within the pore or inhibition of anion flux secondary to compression of the pore by NFA; the occupancy of the pore by anions would either compete with NFA binding or counteract channel pore compression induced by allosteric NFA binding.

NFA ability to suppress current of TMEM16A was used to confirm the involvement of this channel in different biological processes like for example rodent vomeronasal transduction [61], chloroquine gastrointestinal side effects [62], human spermatozoa acrosomal reaction [63]. TMEM16A is also expressed in brain capillary endothelial cells, and it is involved in proliferation and migration of these cells, contributing to blood brain barrier homeostasis. NFA (100 μM) reduced chloride current and consequently cell migration, proliferation and trans‐endothelial permeability, highlighting the potential repurposing of NFA and analogs in diseases associated with blood brain barrier dysfunction [64] (Figure 3). Additionally, NFA inhibition of TMEM16A caused improvement of intestinal motility in the rat model of irritable bowel syndrome (IBS) [65], blocked mucus [66], and salivary production [67] and avoided airway hyperresponsiveness in ovalbumin‐sensitized mice [68], suggesting promises for future application in pathological conditions, including IBS [65], airway mucus secretion [66], chronic asthma [68], and high salivary production [67].

Besides TMEM proteins, bestrophins (BEST1 to BEST4 genes) likely contribute to CaCCs in some cells [69]. Mutations in BEST1 are associated with a spectrum of ophthalmic disorders characterized by retinal degeneration. NFA was shown to inhibit CaCCs in cells transfected with murine Best1, Best2, and Best3, and human Best1 [58, 70, 71, 72]. In a comparative study performed in transfected CHO cells, NFA was more potent in inhibiting TMEM16A (IC_50_ = ~7.5 μM) than Best1 (IC_50_ = ~100 μM) [58]. However, the limited selectivity of NFA between TMEM16 and bestrophin chloride channels might complicate the interpretation of pharmacological studies on native CaCCs.

## 4 CFTR

The cystic fibrosis transmembrane conductance regulator (CFTR) is expressed on the apical membrane of epithelial cells and mediates chloride ion transport across the membranes, regulating electrolytes and fluid flow. Diseases like cystic fibrosis, secretory diarrhea, or autosomal dominant polycystic kidney disease are linked to CFTR mutations [73].

Observing the structural similarity between NFA and arylaminobenzoate diphenylamine‐2‐carboxylate, a CFTR inhibitor, the hypothesis that NFA could act as a CFTR inhibitor was investigated in cardiomyocytes [74] and in colon epithelia [75]. The precise mechanism of NFA inhibition consists in an open‐channel block of CFTR, with a weaker potency compared to CaCCs (IC_50_ = ~250 μM) [76] (Figure 3). Yet, this discovery may open the way to new therapeutic opportunity for diseases with an abnormal increased activity of CFTR channel like autosomal dominant polycystic kidney disease.

Investigation of fenamate effects on T84 cell monolayers, as a model of human intestinal epithelium, showed a greater efficacy of FFA (IC_50_ = ~8 μM) compared to MCFA, MFA, and TFA (IC_50_ = ~23, 26, and 100 μM, respectively) (Figures 1 and 2), in blocking cAMP‐dependent chloride secretion due to inhibition of both apical CFTR chloride channel and basal KCNQ1/KCNE3 potassium channel [77]. Remarkably, the IC_50_ for inhibition of basal potassium current was ~1 μM for FFA. In the same study, fenamates also inhibited Ca^2+^‐dependent chloride secretion (IC_50_ = ~10 μM) supported by apical CaCCs and basal Ca^2+^‐dependent potassium channels (KCa3.1). Such a picture highlights how complex the interpretation of fenamate effects may be due to their pleiotropic effect on ion channels. Altogether, these results suggest a possible mechanism contributing to the constipation side effect of fenamates (especially FFA) and their potential to contrast secretory diarrhea [78]. Importantly, studies on *Xenopus* oocytes demonstrated that high concentration of FFA can inhibit CFTR through direct binding to the channel with a higher efficacy at positive membrane voltage [79] (Figure 1).

## 5 Ligand‐Gated Chloride Channels

Ligand‐gated chloride channels include glycine receptors (GlyRs) and GABA_A_ receptors. GlyRs are mainly expressed in spinal cord, brain stem, cerebellum and retina and are involved in pain sensation, perception of visual, acoustic and sensory signals and movement control. Alterations of their function cause hyperekplexia and temporal lobe seizures with memory deficits [80, 81]. A few studies are available regarding fenamates modulation of this channel. Maleeva and collaborators showed the voltage‐dependent inhibition exerted by NFA on GlyRs expressed in CHO cells by NFA, which depended on the channel subunit composition; major inhibition was found on α2 and α3 subunits (IC_50_ = ~25 μM at +30 mV) compared to α1 GlyRs (IC_50_ = ~100 μM at +30 mV) [82]. These results were corroborated by analyzing NFA effects on glycine triggered inhibitory post‐synaptic currents in mouse hypoglossal motoneurons [83] (Figure 3). Inhibition by NFA was more pronounced on motoneurons from P2 to P4 mice, which are expected to express mainly the neonatal α2 GlyR subunit, than motoneurons from P7 to P12 mice which may express mainly α1 GlyR subunit. In an independent study, the screening of a ~1000‐compound chemical library in a zebrafish embryo model led to the identification of FFA as a possible GlyR inhibitor similarly to the well‐known inhibitor strychnine [84]. In the same study, inhibition of glycine‐mediated currents was confirmed in oocytes transfected with α1 or α1β1 human GlyR subunits.

Several studies have extensively investigated the modulatory activity in vitro and in vivo of fenamates on GABA_A_ receptor. The GABA_A_ receptors are the main chloride channels involved in inhibition of action potential in neuronal cells, which opening is mediated by GABA neurotransmitter binding that causes an influx of Cl^−^ ions and, consequently, membrane hyperpolarization [85]. Former studies showed that MFA had a dual effect on GABA_A_ receptors expressed in *Xenopus laevis* oocytes according to concentration: in the presence of 10 μM of GABA, low concentration of MFA enhanced GABA‐dependent current (EC_50_ = ~5 μM), while it had an opposite inhibitory effect at higher concentrations (IC_50_ = ~30 μM) [86] (Figure 2). This dual effect was later confirmed on native GABA_A_ receptors in isolated rat cerebellar Purkinje cells and was shown to depend also on GABA concentration; thus, the net effect depends on both GABA and fenamate concentrations [87]. This study also suggested the presence of two binding sites for MFA. Inhibition of GABA_A_ by MFA is voltage‐dependent and requires open‐channel block, suggesting binding inside the pore. Diversely, the potentiating site might be located at the transmembrane β(+)/α(−) inter‐subunit interface, being partly shared with other positive allosteric modulators, such as diazepam and etomidate [87]. Yet, the benzodiazepine antagonist flumazenil has no effect on GABA_A_ potentiation by fenamate [86].

Fenamate effects also depend on the presence of different β subunits in the receptor complex: β2 or β3 subunits confer an agonistic effect of MFA on GABA_A_ receptor, whereas the presence of β1 subunit was associated with an inhibitory response. Specifically, the amino acid in position 290 within the second transmembrane domain (asparagine in β2 and β3 [N290] or serine in β1 [S290]) was critically involved in fenamate effect [88]. This was corroborated by in silico studies [87]. The pivotal role of β subunits in fenamates modulation was further confirmed in mouse Ltk^−^ fibroblast cells expressing recombinant human GABA_A_ subunits; MFA, as well as FFA, MCFA, TFA, and NFA were able to potentiate GABA_A_ receptors containing β2 or β3 subunits with different efficacy (Figures 1, 2, 3). By contrast, all fenamates had an inhibitory effect on α1–β1–γ2 receptor [89]. Agonistic effects of fenamates were confirmed in native GABA_A_ receptors in rat embryonic hippocampal neurons, with MFA > TFA > MCFA = FFA > NFA order of potency [90], and in neurons differentiated from human stem cell lines [91].

The allosteric potentiation of GABA neurotransmission might contribute to the known anti‐epileptic and neuroprotective effects of fenamates, while overdose effects include seizures and coma. In addition, as GABA_A_ receptors participate in pain transmission, NFA has been proposed as a drug for the treatment of neuropathic pain. Indeed, NFA can reduce the primary afferent depolarization by enhancing presynaptic GABA‐A receptor mediated inhibition. This action decreases excitatory neurotransmitter release from sensory afferents, thereby attenuating pain signaling in neuropathic conditions [92].

Taken together, these data illustrated the modulatory activity of fenamates on GABA_A_ receptor, suggesting possible clinical application and their attractive potential for the development of more selective derivatives [93].

## 6 Conclusion

This review has shown that fenamates have additional targets beyond cyclooxygenases, whose inhibition allows them to exert their traditional anti‐inflammatory effect. Among the emerging non‐COX targets, chloride channels are considered highly promising due to their involvement in many pathological conditions and to their potential for drug targeting. Fenamates have long been used as pharmacological tools to study ion channels, especially chloride channels, in order to verify their involvement in cell function and to characterize their biophysics. However, in most cases, the molecular mechanisms underlying fenamates effects on these channels are still unclear. In addition, because of the limited selectivity of fenamates, caution should be paid to the interpretation of the results. A way to overcome such a limit may consist of the use of several channel modulators to better define the ion channel isoforms involved in the studied cell function.

Although the modulation of chloride channel activity occurs at higher concentrations compared to COX inhibition, fenamate plasma concentration in the clinical setting may reach levels able to inhibit a number of chloride channels, suggesting that their inhibition may contribute to the therapeutic or toxic effects of NSAIDs [94, 95]. Accordingly, fenamates have acquired increasing importance for their potential *repurposing* in pathological conditions characterized by chloride channel disfunction including neuromuscular diseases, epilepsy, IBS, chronic asthma, secretory diarrhea, and neuropathic pain. For instance, to date there are ongoing clinical trials that are exploring the effects of fenamates in combination with other drugs for the treatment of psychotic disorders (NCT029930005), brain metastasis (NCT02429570) and glioblastoma (EudraCT 2021‐000708‐39).

Overall, this review highlights the modulatory activity of fenamates on different classes of chloride channel, describing multiple effects as agonistic and antagonistic molecules based on isoforms, drug concentrations, and cellular/tissue types. Notably, the efficacy of these compounds exhibits a voltage‐dependent inhibitory activity, with remarkably higher effect at depolarized potential, which may help reconcile disparities in reported IC_50_ values. The pleiotropic activity of fenamates offers multiple attractive applications but may also imply many off‐target and side effects. First of all, it is well known that fenamates may induce gastrointestinal, kidney, and cardiovascular side effects due to COX inhibition, especially after prolonged use. Thus, fenamates side effects may hamper their repositioning for the treatment of chronic diseases, including chloride channelopathies. On the other hand, fenamates may serve as lead compounds for docking studies and for the development of new derivatives with improved activity and ion channel selectivity to avoid or minimize adverse and off‐target events.

In conclusion, among the NSAIDs, fenamates represent one of the most attractive drugs able to modulate chloride channels and may serve as a paradigm for future development of novel drugs for chloride channelopathies.

## 7 Nomenclature of Targets and Ligands

Key protein targets and ligands in this article are hyperlinked to corresponding entries in http://www.guidetopharmacology.org, the common portal for data from the IUPHAR/BPS Guide to PHARMACOLOGY [96], and are permanently archived in the Concise Guide to PHARMACOLOGY 2023/24: Ion channels [97].

## Author Contributions

**Paola Laghetti:** conceptualization, writing – original draft, funding acquisition. **Ilaria Saltarella:** conceptualization, writing – original draft, funding acquisition. **Simone Dell'Atti:** conceptualization, writing – original draft, funding acquisition. **Jean‐François Desaphy:** conceptualization, writing – review and editing, supervision. **Concetta Altamura:** conceptualization, funding acquisition, writing – review and editing, supervision.

## Conflicts of Interest

The authors declare no conflicts of interest.

## Data Availability

The authors have nothing to report.
